# The outcome and prognostic factors for lymph node recurrence after node-sparing definitive external beam radiotherapy for localized prostate cancer

**DOI:** 10.1186/s12957-015-0721-4

**Published:** 2015-11-06

**Authors:** Yu-Jen Wang, Chao-Yuan Huang, Wei-Hsien Hou, Chia-Chun Wang, Keng-Hsueh Lan, Chung-Hsin Chen, Hong-Jen Yu, Ming-Kuen Lai, Ann-Lii Cheng, Shihh-Ping Liu, Yeong-Shiau Pu, Jason Chia-Hsien Cheng

**Affiliations:** Department of Radiation Oncology, Taipei Medical University-Shuang Ho Hospital, New Taipei City, Taiwan; Division of Radiation Oncology, College of Medicine and Hospital, National Taiwan University, No. 7 Chung-Shan South Road, Taipei, 10002 Taiwan; Departments of Oncology, College of Medicine and Hospital, National Taiwan University, Taipei, Taiwan; Departments of Urology, College of Medicine and Hospital, National Taiwan University, No. 7 Chung-Shan South Road, Taipei, 10002 Taiwan; Graduate Institutes of Oncology, College of Medicine, National Taiwan University, Taipei, Taiwan; Clinical Medicine, College of Medicine, National Taiwan University, Taipei, Taiwan

**Keywords:** Prostate cancer, Radiotherapy, Lymph node, Recurrence, Prognosis

## Abstract

**Background:**

The prognostic factors for the recurrence of lymph node (LN) metastasis after dose-escalated radiotherapy (RT) in prostate cancer patients have not been well investigated. We report the prognostic factors and outcomes in patients receiving salvage treatment for LN recurrence after high-dose intensity-modulated RT (IMRT).

**Methods:**

We studied a cohort of 419 patients with localized prostate adenocarcinoma undergoing definitive IMRT (78 Gy). LN recurrence was diagnosed by size criteria using computed tomography (CT) or magnetic resonance imaging, or abnormal uptake of ^18^F-fluorocholine by LNs on positron emission tomography/CT. Overall survival and LN recurrence-free survival (LNRFS) were calculated, and prognostic factors were evaluated.

**Results:**

With a median follow-up of 60 months, 18 patients (4.3 %) had LN recurrence and a significantly lower 5-year overall survival rate (60 vs. 90 %, *p* = 0.003). Univariate analysis showed that T3/T4 stage (*p* = 0.003), Gleason score >7 (*p* < 0.001), and estimated risk of pelvic LN involvement of >30 % by the Roach formula (*p* = 0.029) were associated with significantly lower LNRFS. On multivariate analysis, high Gleason score (hazard ratio = 5.99, *p* = 0.007) was the only independent factor. The 1/2-year overall survivals after LN recurrence were 67/54 %. Patients with isolated LN recurrence (*p* = 0.003), prostate-specific antigen (PSA) doubling time >5 months (*p* = 0.009), interval between PSA nadir and biochemical failure >12 months (*p* = 0.035), and PSA <10 ng/ml at LN recurrence (*p* = 0.003) had significantly better survival. Patients with isolated LN recurrence had significantly better survival when treated with combined RT and hormones than when treated with hormones alone (*p* = 0.011).

**Conclusions:**

Gleason score of >7 may predict LN recurrence in prostate cancer patients treated with definitive IMRT. Small number of patients limits the extrapolation of this risk with the primary treatment strategy. Combined RT and hormones may prolong survival in patients with isolated LN recurrence.

## Background

Definitive radiotherapy (RT) is a first-line treatment for patients with localized prostate cancer (PCa) [1, 2]. Dose-escalated intensity-modulated radiation therapy (IMRT) has been increasingly used in clinical practice for PCa [3] and results in satisfactory outcomes and acceptable toxicity [4–6]. Compared with three-dimensional conformal radiation therapy, IMRT permits delivery of higher radiation dose while reducing the risk of gastrointestinal toxicity [7–9]. Most dose-escalated radiotherapies are designed to treat the prostate and seminal vesicles but not the pelvic lymphatics. Although prophylactic irradiation of lymph nodes has long been recommended for head and neck cancer, rectal cancer, anal cancer, breast cancer, and certain solid tumors, the therapeutic ratio of whole pelvic RT remains unknown for PCa. Randomized trials have failed to provide solid evidence for the survival benefit of additional RT to the pelvic lymphatics [10–12].

The lymph node is the second most common metastatic site in prostate cancer [13]. The nomogram to predict lymph node involvement for prostate cancer was developed using known prognostic factors, including pretreatment prostate-specific antigen (PSA), Gleason score, and clinical stage as the key factors for estimating the risk of lymph node metastasis [14, 15]. Although the percentage of lymph node metastasis is low in PCa patients undergoing radical prostatectomy, lymph node dissection is known to prolong their survival [16, 17]. However, the prognostic factors that predict the recurrence of lymph node metastasis in PCa patients (after definitive RT directed only to prostate and seminal vesicles) has not been well investigated.

For PCa patients with lymph node recurrence, androgen deprivation therapy (ADT) is the treatment of choice [1, 2]. The outcomes for PCa patients with lymph node metastasis vary between studies. Some studies report relatively good outcome in patients with lymph node recurrence receiving aggressive salvage treatment [18–20]. In accordance with a trial of dose-escalated RT at our institution, we have been using definitive IMRT (78 Gy) for PCa patients since 2003, targeting only the prostate and involved seminal vesicles. In this study, our aim was to determine the factors predicting treatment failure by investigating the characteristics of patients with lymph node recurrence after definitive RT without elective pelvic node irradiation and their outcomes with salvage treatment.

## Methods

### Patients and diagnostic criteria for lymph node metastasis

The cohort consisted of 419 Asian patients with non-metastatic prostate adenocarcinoma treated in our institute from December 2003 to December 2010. The study has been approved by the ethical committees related to the institution in which it was performed, and patients gave informed consent to the study. All patients underwent initial treatment with either step-and-shoot IMRT, tomotherapy, or volumetric modulated arc therapy. In all patients, the RT dose was 78 Gy in 39 fractions, and the clinical target volume (CTV) consisted of the prostate and the involved seminal vesicle(s) but not the pelvic lymph nodes. The initial treatment with ADT was left to the discretion of the prescribing physicians. ADT was administered neoadjuvantly more than 2 months prior to RT and continued concurrently with RT. Alternatively, maintenance ADT was administered concurrently with RT and was continued after RT. Patients typically received gonadotropin-releasing hormone (GnRH) agonist as monotherapy. An oral anti-androgen was usually initiated at the start of GnRH agonist therapy to prevent a rebound surge of androgen.

Biochemical failure after the primary RT was defined according to the Phoenix definition (PSA elevation exceeding PSA nadir by 2 ng/ml). The diagnosis of lymph node metastasis was based on the following criteria: the short-axis diameter of the lymph node was elongated and exceeded 10 mm or was rounded and exceeded 8 mm on computed tomography (CT) or magnetic resonance imaging (MRI) or abnormal uptake by lymph nodes of ^18^F-fluorocholine on positron emission tomography (PET)/CT. PSA doubling time was defined as the time interval needed for serum PSA levels to increase by 100 %. The PSA nadir–biochemical failure interval was defined as the interval between the lowest PSA level and the diagnosis of biochemical failure.

### Follow-up and toxicity related to salvage treatment

Follow-up duration, survival time, and event time were calculated from the start of the initial IMRT for the comparison between patients with and without lymph node recurrence and from the start of salvage treatment only for patients with lymph node recurrence. Kaplan-Meier analysis was performed to determine overall survival and lymph node recurrence-free survival rates. Treatment-related toxicities were determined using Common Toxicity Criteria v.4.0.

### Statistical analysis

Descriptive analysis was performed by calculating ranges, means, medians, and standard deviations. Continuous variables were compared with a two-sided unpaired *t* test. Chi-square and Fisher’s exact tests were used for contingency table analysis. The log-rank test was used to determine prognostic factors affecting survival. All prognostic variables found to be significant or borderline significant in univariate analysis were included in multivariate analysis using the Cox proportional hazards regression model. Significance was assumed if *p* < 0.05. All statistics were done with PASW Statistics 18 (IBM Corp., Armonk, NY, USA).

## Results

### Differences in characteristics between patients with and without lymph node recurrence

With a median follow-up of 60 months, 18 of 419 patients (4.3 %) had lymph node recurrence. Of these 18 patients, 3 patients had initial T1, 4 had T2, 9 had T3, and 2 had T4 disease. Eleven patients (61.1 %) had a Gleason score of 8 or higher. The median value of initial PSA was 18.4 ng/ml (range 4.8–101.5). Sixteen patients (88.8 %) had high risk or very high risk of PCa according to the National Comprehensive Cancer Network (NCCN) classification. Their characteristics are shown in Table 1.

**Table 1:** Patient/tumor characteristics and the use of androgen deprivation therapy (ADT) between patients without (*n* = 401) and with (*n* = 18) lymph node (LN) recurrence

Variable	Without LN	With LN
	Number (%)	Number (%)
Age		
<70	134 (33.4)	5 (27.7)
≥70	267 (66.6)	13 (72.3)
T stage		
1a	5 (1.2)	0 (0)
1b	6 (1.5)	0 (0)
1c	124 (30.9)	3 (16.7)
2a	52 (13.0)	1 (5.6)
2b	40 (10.0)	3 (16.7)
2c	42 (10.5)	0 (0)
3a	72 (18.0)	2 (11.1)
3b	53 (13.2)	7 (38.9)
4	7 (1.7)	2 (11.1)
Gleason score		
<7	125 (31.2)	3 (16.7)
7	185 (46.1)	4 (22.2)
8–10	91 (22.7)	11 (61.1)
PSA (ng/ml)		
<10	117 (29.2)	4 (22.2)
10–20	119 (29.7)	5 (27.7)
≥20	165 (41.1)	9 (50.0)
Risk group		
Low	41 (10.2)	1 (5.6)
Intermediate	121 (30.2)	1 (5.6)
High	180 (44.9)	7 (38.9)
Very high	59 (14.7)	9 (50.0)
Any ADT		
No	57 (14.2)	2 (11.1)
Yes	344 (85.8)	16 (88.9)
Neoadjuvant ADT		
No	127 (31.7)	4 (22.2)
Yes	274 (68.3)	14 (77.8)
Concurrent ADT		
No	103 (25.7)	8 (44.4)
Yes	298 (74.3)	10 (55.6)
Maintenance ADT		
No	177 (44.1)	9 (50.0)
Yes	224 (55.9)	9 (50.0)

**Fig. 1:**
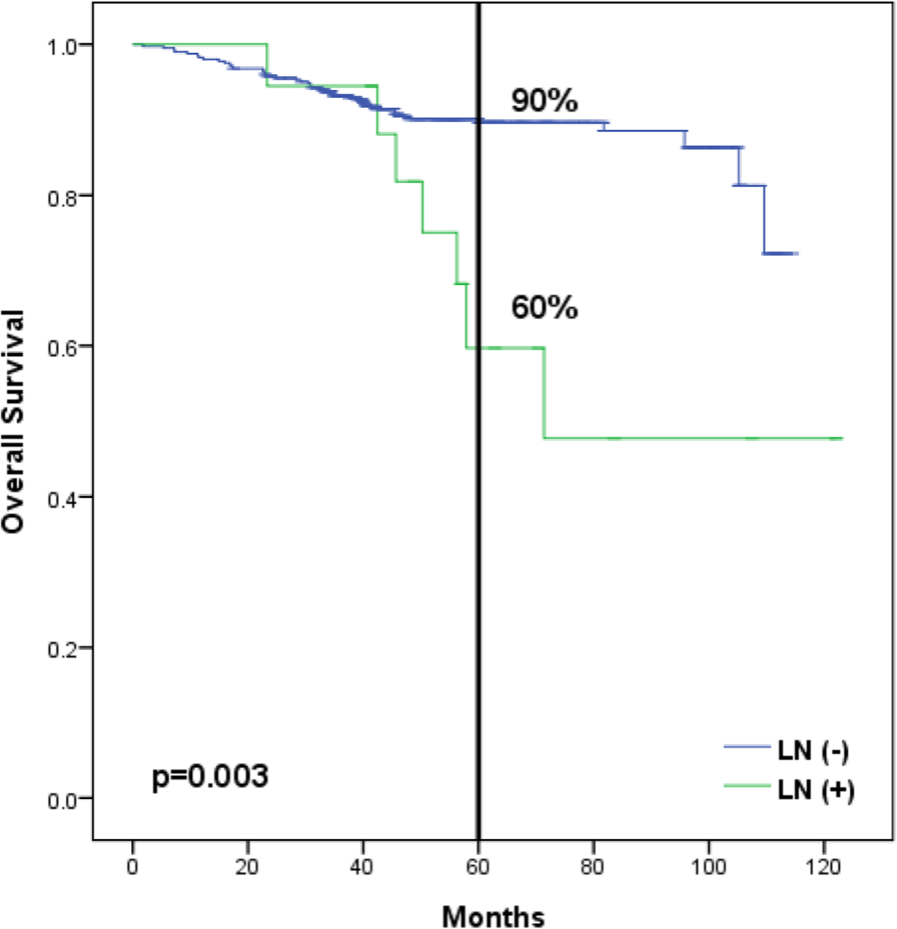
Kaplan-Meier analyses of overall survival of 419 prostate cancer patients without (401 patients) and with (18 patients) lymph node (*LN*) recurrence

As shown in Fig. 1, the 5-year overall survival rate was significantly lower in patients with lymph node recurrence (60 vs. 90 %, *p* = 0.003). Univariate analysis identified advanced T stage (T3/T4 vs. T1/T2) (*p* = 0.003), high Gleason score (>7 vs. ≤7) (*p* < 0.001), and the estimated risk of pelvic lymph node involvement by the Roach formula of more than 30 % (*p* = 0.029) as factors associated with higher risk of lymph node recurrence. On multivariate analysis, high Gleason score (hazard ratio = 5.99, *p* = 0.007) was the only independent prognostic factor for lymph node recurrence, while advanced T stage was of borderline significance (hazard ratio = 2.68, *p* = 0.074) (Table 2).

**Table 2:** Univariate and multivariate analyses of the prognostic factors on lymph node recurrence-free survival

Variable	5-year lymph node recurrence-free rate (%)	*p* value	HR (95 % CI)	*p* value
Age				
≥70	96	0.972		
<70	97			
T stage				
T3–T4	93	0.003	2.68 (0.91–7.90)	0.074
T1–T2	98			
Gleason score				
8–10	98	<0.001	5.99 (1.62–22.14)	0.007
≤7	90			
PSA (ng/ml)				
≥30	94	0.100	1.26 (0.33–4.82)	0.74
<30	97			
RFLN (%)				
>30	93	0.029	0.51 (0.10–2.60)	0.419
<30	98			
ADT				
Yes	96	0.732		
No	96			

*ADT* Androgen deprivation therapy, *CI* confidence interval, *HR* hazard ratio, *RFLN* Roach formula for the risk of pelvic lymph node metastasis

### Salvage treatment in patients with lymph node recurrence

Of the 18 patients with lymph node recurrence, 13 patients had isolated lymph node recurrence, defined as any recurrence at nodal regions without other distant metastasis. Eight patients had pelvic lymph node recurrence alone (N1); 2 had simultaneous pelvic and para-aortic lymph node recurrences (M1a); and 3 had neck, axilla, and anterior mediastinum lymph node recurrences (M1a). The median time from primary radiation therapy to biochemical failure was 36 months (range 15–99). Recurrence in 9 patients was diagnosed by both ^18^F-fluorocholine PET/CT and MRI, 8 by CT and 1 by MRI. At the diagnosis of lymph node recurrence, the median age was 71.5 years, the median PSA level was 20.0 ng/ml (range 2.0–680), and the median PSA nadir-failure interval was 16.7 months (range 5.9–76.2). All patients with lymph node recurrence received salvage hormone treatment. Of the 13 patients with isolated lymph node recurrence, 6 patients underwent additional RT with a median dose of 55 Gy (range 55–63) to the involved lymph nodes. All 6 patients had a normal PSA level after salvage RT for a median of 17 months. One patient with isolated lymph node recurrence underwent salvage pelvic lymph node dissection, had a second biochemical failure, and received salvage RT resulting in PSA control. Six patients with isolated lymph node recurrence received hormone therapy alone, with 4 of them experiencing disease progression. One of them with initial pelvic lymph node recurrence had subsequent para-aortic lymph node recurrence and received additional salvage RT.

### Survivals and prognostic factors after salvage treatment for lymph node recurrence

With a median follow-up of 12 months from the salvage treatment, 7 patients died, with 6 of them due to prostate cancer. Relapse of lymph node metastasis occurred after treatment in one patient and subsequently to other lymph nodes in 11 patients; 5 patients had bone metastasis. The 1- and 2-year overall survival rates after lymph node recurrence were 67 and 54 %, respectively, with a median survival of 12.3 months. None of the 18 patients had more than grade 2 toxicity related to the salvage treatments. Patients with isolated lymph node recurrence (*p* = 0.003), PSA doubling time >5 months (*p* = 0.009), PSA nadir-to-biochemical failure interval >12 months (*p* = 0.035), and PSA <10 ng/ml at the diagnosis of lymph node recurrence (*p* = 0.003) had significantly better overall survival. On multivariate analysis, none of these prognosticators proved to be of significance (Table 3).

**Table 3:** Univariate and multivariate analyses of the prognostic factors on overall survival after lymph node recurrence

	2-year survival rate (%)	*p* value	HR (95 % CI)	*p* value
Age				
≥70	41	0.160		
v<70	80			
T stage				
T3–T4	37	0.236		
T1–T2	100			
Gleason score				
8–10	47	0.661		
≤7	63			
PSA (ng/ml)				
≥20	50	0.762		
<20	58			
Isolated lymph node recurrence				
Yes	67	0.003	5.22 (0.50–55.03)	0.169
No	0			
PSA doubling time				
>5 months	89	0.009	1.869 (0.05–74.19)	0.739
<5 months	17			
PSA nadir-free interval				
>12 months	80	0.035	1.203 (0.09–15.62)	0.888
<12 months	20			
PSA at lymph node recurrence (ng/ml)				
>10	19	0.003	510,993 (0–1.22E184)	0.950
<10	100			

*ADT* Androgen deprivation therapy, *HR* hazard ratio, *CI* confidence interval

### Patients with isolated lymph node recurrence treated with combined RT and hormone therapy

**Fig. 2:**
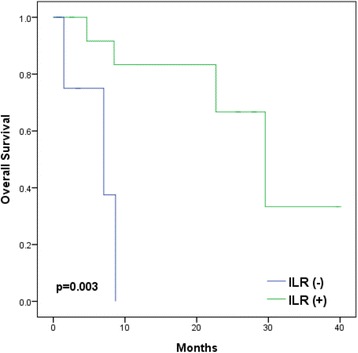
Kaplan-Meier analyses of overall survival of 18 prostate cancer patients with isolated lymph node recurrence (*ILR*) (13 patients) and with simultaneous LN recurrence and distant metastasis (5 patients)

**Fig. 3:**
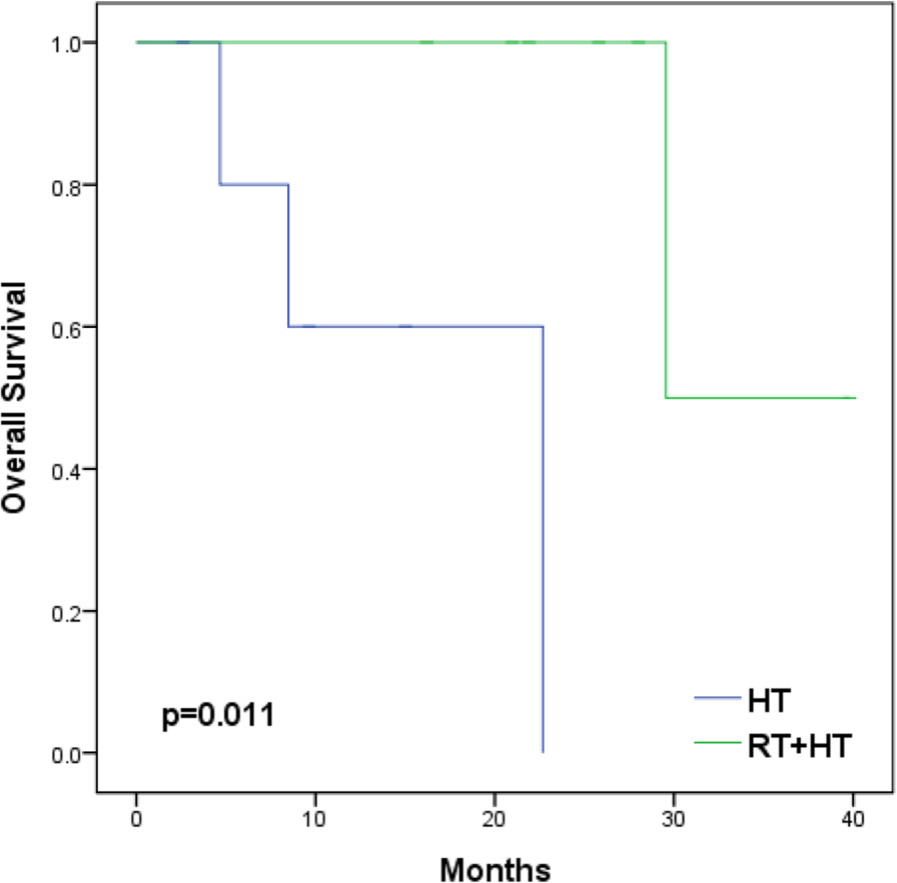
Kaplan-Meier analyses of overall survival of 13 prostate cancer patients with exclusive lymph node recurrence treated with combined radiotherapy and hormone therapy (*RT + HT*) (seven patients) or hormonal therapy alone (*HT*) (six patients)

We compared the survival rates between patients with isolated lymph node recurrence (13 patients) and with simultaneous lymph node recurrence and distant metastasis (5 patients). The 1- and 2-year survival rates were 83/67 and 0/0 % (*p* = 0.003), respectively (Fig. 2). For the 13 patients with isolated lymph node recurrence treated with combined RT and hormone therapy (7 patients) or hormonal therapy alone (6 patients), the 1- and 2-year survival rates were 100/100 % (median survival 25.8 months) and 60/0 % (median survival 9.0 months), respectively (*p* = 0.011) (Fig. 3).

## Discussion

Little is known about the risk factors of lymph node recurrence in PCa patients after definitive RT as well as their outcome and prognosis after salvage treatment for nodal recurrence, especially in the era of dose-escalated RT. Our current study is the first to focus on the lymph node recurrence in PCa patients undergoing the 78-Gy IMRT to the prostate and involved seminal vesicles but not the pelvic nodes. We identified not only characteristics associated with lymph node recurrence but also the prognostic factors determining outcome in patients with nodal recurrence treated by salvage treatment. Moreover, we showed that RT combined with hormones had great therapeutic value in patients with isolated lymph node recurrence.

Lymph node metastasis, though infrequent, has been recognized as a negative prognostic factor of PCa, and its risk of occurrence has been associated certain tumor characteristics. In our series, the 5-year overall survival rate was significantly lower in patients with lymph node recurrence. Previous studies have investigated the probability of pelvic lymph node involvement using the established predictive nomogram [14, 15]. Gleason sum and PSA level were found to predict the risk of lymph node involvement for post-prostatectomy patients [15]. Our study (which excluded patients receiving elective pelvic node irradiation) similarly identified Gleason sum as the only independent factor associated with lymph node recurrence. In contrast, the usefulness of the Roach formula as a tool for predicting lymph node metastasis in contemporary patients is debatable [21, 22]. With bowel-related quality of life being better in PCa patients receiving prostate-only RT than whole-pelvis RT [23], the role of prophylactic lymphatic RT remains unsettled. For the high-risk PCa patients, questions about the effects of pelvic nodal irradiation are being addressed by the ongoing clinical trial (RTOG 0924) [24].

Of note, both N1 and M1a in the current staging system are referred to as lymph node metastasis and may infer a diversity of outcomes. The salvage treatments and survival rates of PCa patients with lymph node recurrence were very heterogeneous [25, 26]. Therefore, prognostic factors predictive of lymph node recurrence would be expected to help doctors tailor treatment to achieve the best possible outcome. Pond et al. reported that median survival was longest in patients with lymph node-only disease (26.7 months), followed by bone-only and bone-plus-node disease [25]. Yossepowitch et al. found that longer PSA doubling time is a favorable prognostic factor in post-prostatectomy patients [26]. Moreover, our univariate analysis identified isolated lymph node recurrence, PSA nadir-to-biochemical failure interval, and PSA level at the diagnosis of lymph node recurrence, in addition to PSA doubling time, as significant prognostic factors. Modern imaging tools, such as ^18^F-fluorocholine PET/CT, have increased the detectability of small and otherwise undetectable lymph node metastases and thus may increase PCa prevalence at even lower PSA levels [27–29].

Salvage ADT was given to all our patients as the suggested treatment guideline [2]. Nevertheless, ADT is not a curative treatment, with most treated patients ultimately developing castration-resistant disease [30]. Two studies used an advanced RT technique (stereotactic body radiation therapy) as a salvage modality and achieved safe and effective local control [18, 31], while other studies performed salvage lymph node dissection and obtained a certain degree of biochemical response [20, 32]. Additionally, adjuvant RT had a beneficial impact on survival in pN1 PCa patients with low-volume intermediate-/high-grade nodal disease and intermediate-volume nodal disease [33]. Metastasis-directed therapy was recommended as a promising approach with acceptable toxicity for PCa patients with oligometastasis, mostly with lymph node metastasis [34]. With isolated LN recurrence representing limited volume of disease, our series similarly showed that combining RT and hormones (rather than treating with hormones alone) significantly increased survival. The 2-year survival rate of 100 % and the median survival of 25.8 months were encouraging for this select group of patients treated with combined modalities including local RT and systemic hormones.

Unlike surgery, which usually provide nodal dissection along with radical prostatectomy, RT is more commonly delivered to prostate and seminal vesicles only. The difference in primary nodal treatment between the two modalities is reflected in the outcomes of patients with lymph node metastasis. The 5-year survival rate of 96 % was shown in a Chinese series of PCa patients with lymph node metastasis on radical prostatectomy [35], compared with 60 % in our patients treated by node-sparing RT. However, the small number of patients (18) and the low crude rate (4.3 %) of lymph node metastasis from the whole group in this study limit the extrapolation of the risk of lymph node metastasis with the primary treatment strategy including pelvic RT. Besides RT, hormonal therapy has been the effective treatment to improve the survival outcomes of PCa patients with adverse characteristics, such as high Gleason score [36].

Our series has limitations. First of all, our data included only a small number of patients with lymph node recurrence, even after a long follow-up period. The small patient number was likely to be the reason that no significant prognostic factor was identified on multivariate analysis of survival after lymph node recurrence. Ideally, this limitation might be solved by future multi-center studies. Secondly, the retrospective nature of the study and the uncontrolled use of hormone therapy in the primary treatment might bias estimates of the true prevalence of lymph node recurrence. Finally, the heterogeneity of salvage treatments given after the lymph node recurrence unavoidably confounded the prognosis. Further prospective study with longer follow-up time is needed.

## Conclusions

In conclusion, our data of PCa patients treated with definitive IMRT and not elective pelvic node irradiation indicated the significant association of high Gleason score with lymph node recurrence. Isolated lymph node recurrence, PSA doubling time of >5 months, PSA nadir-to-biochemical failure interval of >12 months, and PSA of <10 ng/ml at the diagnosis of lymph node recurrence were the factors associated with better overall survival after biochemical failure. For patients with isolated LN recurrence, the combined use of RT to the involved nodes and hormones resulted in the longer survival.
